# Influence of foot strike pattern on co‐contraction around the ankle and oxygen uptake during running at 19 km/h

**DOI:** 10.14814/phy2.70023

**Published:** 2024-09-08

**Authors:** Shimpei Kubo, Katsutoshi Yaeshima, Takahito Suzuki, Eiji Daigo, Yu Kitaoka, Ryuta Kinugasa

**Affiliations:** ^1^ Department of Human Science Kanagawa University Yokohama Japan; ^2^ Department of Welfare and Culture Okinawa University Okinawa Japan

**Keywords:** coactivation, forefoot strike, rearfoot strike, running economy

## Abstract

This study investigated the coactivation of plantar flexor and dorsiflexor muscles and oxygen uptake during running with forefoot and rearfoot strikes at 15 and 19 km/h. We included 16 male runners in this study. The participants ran each foot strike pattern for 5 min at 15 and 19 km/h on a treadmill. During the running, respiratory gas exchange data and surface electromyographic (EMG) activity of the medial gastrocnemius (MG), lateral gastrocnemius (LG), soleus, and tibialis anterior muscles of the right lower limb were continuously recorded. The indices of oxygen uptake, energy expenditure (EE), and muscle activation were calculated during the last 2 min in each condition. During the stance phase of running at 15 and 19 km/h, activation of the tibialis anterior and MG muscles was lower and higher, respectively, with forefoot strike than with rearfoot strike. The foot strike pattern did not influence the oxygen uptake. These results suggest that the foot strike pattern has no clear effect on the oxygen uptake when running at 15 and 19 km/h. However, forefoot strike leads to plantar flexion dominance during co‐contraction of the tibialis anterior and MG muscles, which are an antagonist and agonist for plantar flexion, respectively, during the stance phase.

## 1 INTRODUCTION

The foot strike pattern is a possible factor influencing the running economy. Foot strike patterns are conventionally divided into two main categories: forefoot and rearfoot strikes. Several previous studies have reported that oxygen uptake is lower during rearfoot running than during non‐rearfoot running (Gruber et al., 2013; Melcher et al., 2017; Moore et al., 2019; Ogueta‐Alday et al., 2014). For example, running with a rearfoot strike leads to approximately 2% lower oxygen consumption across forefoot and rearfoot strikers at 14.4 km/h compared with running with a forefoot strike (Gruber et al., 2013). However, it is debatable whether the superiority of a rearfoot strike is universal at any speed. For example, it was reported that forefoot strikers ranked relatively high in a marathon (Kasmer et al., 2013). Several athletic coaches assume that rearfoot strikes lead to worse long‐distance performance than forefoot strikes (Abran et al., 2022). The point of foot contact shifts forward with increasing running speeds to approximately 22 km/h (Breine et al., 2014), although the influence of speed ranging up to 18 km/h on the contact point is debatable (Ekizos et al., 2021). The proportion of rearfoot strikers is low in men's 800‐m racing (Hayes & Caplan, 2012). The abovementioned studies have suggested that the forefoot strike is preferable at speeds of elite marathoners and over. However, it has been pointed out that evidence for the influence of foot strike patterns at >18 km/h is insufficient (Ekizos et al., 2021). In fact, the aforementioned studies have reported the superiority of rearfoot strike in a running economy at <15 km/h. Therefore, studies on the high speeds at which runners spontaneously select the forefoot strike are required for the practical application of the foot strike technique.

A difference in the foot strike pattern is derived from different muscle activations. In particular, the activities of the tibialis anterior (TA) (Anderson et al., 2020; Ervilha et al., 2017; Giandolini et al., 2013; Jafarnezhadgero et al., 2021; Landreneau et al., 2014; Lin et al., 2021; Olin & Gutierrez, 2013; Pires et al., 2019; Shih et al., 2013; Valencia et al., 2022; Yong et al., 2014), medial gastrocnemius (MG) (Ervilha et al., 2017; Kelly et al., 2018; Kovács et al., 2023; Landreneau et al., 2014; Lin et al., 2021; Pires et al., 2019; Valencia et al., 2022; Yong et al., 2014, 2020), and lateral gastrocnemius (LG) (Giandolini et al., 2013; Valencia et al., 2022; Yong et al., 2014, 2020) muscles during running with a non‐rearfoot strike are different from those during running with a rearfoot strike. Several studies have found a difference in the activation of the soleus (Sol) muscle (Ervilha et al., 2017; Yong et al., 2014, 2020), which is a synergist of the MG and LG muscles as plantar flexor muscle, between foot strike patterns while others have not (Kelly et al., 2018; Landreneau et al., 2014; Lin et al., 2021; Pires et al., 2019; Yong et al., 2020). A meta‐analysis has demonstrated that Sol muscle activation during running with a non‐rearfoot strike is lower than that during running with a rearfoot strike (Anderson et al., 2020). These electromyographic (EMG) studies dealt with speeds of ≤16 km/h. In addition, the Achilles tendon, which connects to the gastrocnemius and Sol muscles, was longer at the forefoot strike than at the rearfoot strike at 18 km/h, although this difference was not found at ≤14 km/h (Suzuki et al., 2019). The influence of foot strike pattern selection on muscle activities and resultant running economy at elite marathoners' speeds can differ from that at lower speeds. Therefore, we investigated the activation of the plantar flexor and dorsiflexor muscles and oxygen uptake during running with forefoot and rearfoot strikes at 15 and 19 km/h.

## 2 MATERIALS AND METHODS

### 2.1 Participants

We included 16 male runners (age, 19.2 ± 0.9 years; height, 168.8 ± 5.0 cm; weight, 54.5 ± 3.1 kg; mean ± standard deviation) in this study. A priori power analysis (G*Power 3.1.9.6, Dusseldorf, Germany) utilizing an effect size of Cohen's *d* = 0.4 (Gruber et al. 2013), *α* = 0.05, and 1 − *β* = 0.8 indicated that a minimum of 10 participants were required. Therefore, the sample size was deemed sufficient. All participants were well‐trained long‐distance runners who reported running 150–180 km per week. Their performance record in official races for 5000 m was 14:17 ± 0:22 mm:ss. Each participant provided written informed consent according to a protocol approved by the Human Research Ethics Committee at Kanagawa University (2019‐10‐6), which was performed in accordance with the tenets of the Declaration of Helsinki.

### 2.2 Experimental protocol

**FIGURE 1:**
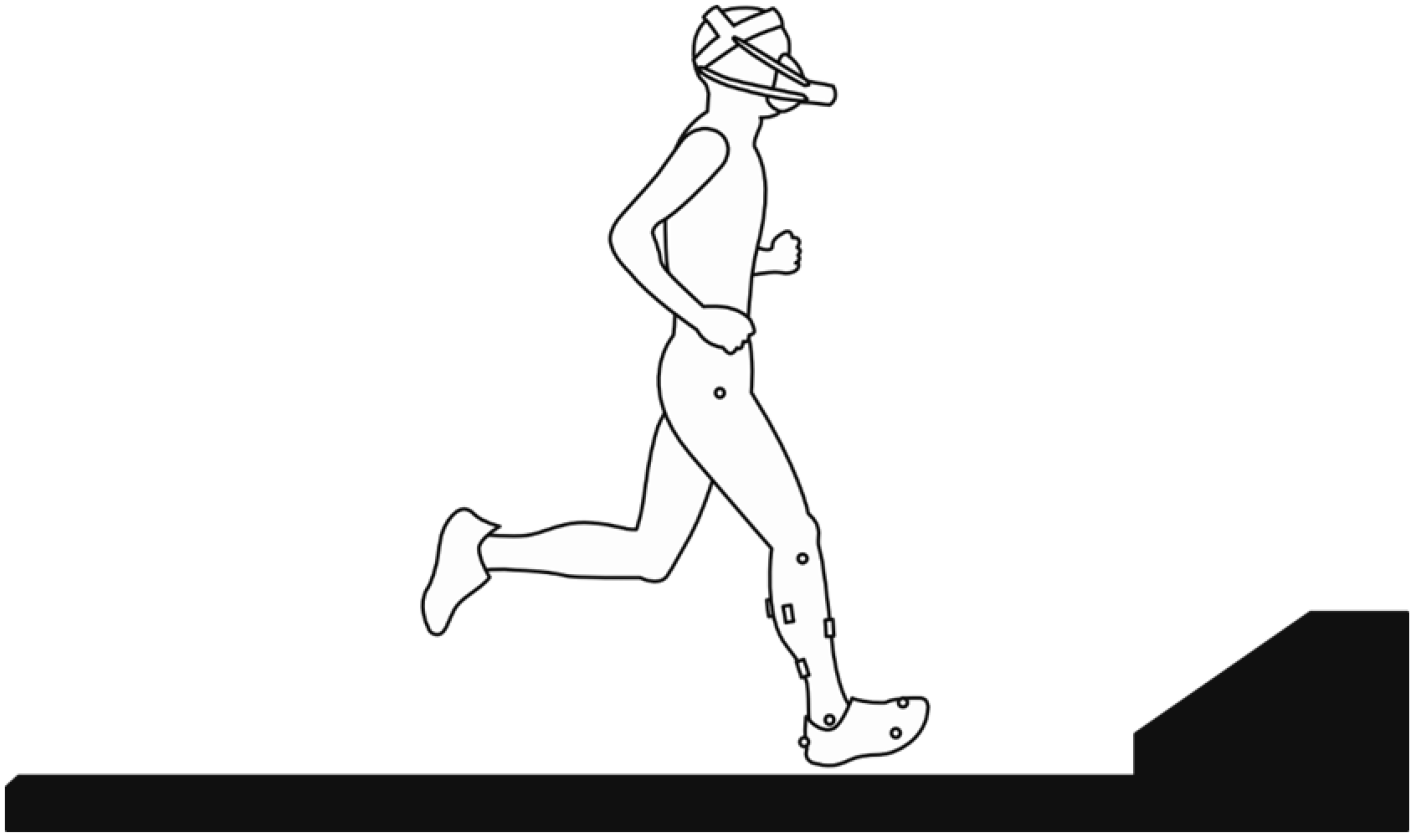
Experimental setup. Squares and circles on the right lower limb with a shoe are the electrodes and reflective markers, respectively.

Each participant attended two laboratory sessions separated by at least 2 days. During the first laboratory session, all participants were familiarized with the unpreferred foot strike technique while running on a motorized treadmill (HTB‐0821, Ohtake‐Root Kogyo, Japan). As a rearfoot strike during running could potentially cause discomfort during the initial runs, familiarization was required. The participants were given several minutes to warm up on a treadmill at their self‐selected pace and preferred foot strike pattern. Subsequently, they ran with a rearfoot strike pattern at 15 km/h followed by 19 km/h for 3 min each, with several minutes of rest. This duration was selected based on participants' training experiences and a previous treadmill familiarization study reporting that even the kinematics of novice treadmill runners were reliable between 2 and 10 min from the start of treadmill running (Lavcanska et al., 2005). During the familiarization, the runners' feet were recorded using a smartphone (Pixel4a, Google, Mountain View, CA, USA) in a high‐speed mode (120 frames/s). The smartphone was placed parallel to the running direction. Both subjective (i.e., rating of perceived exertion, RPE) and objective measures were employed to ensure participant comfort with the running technique. Participants were asked to confirm their comfort levels verbally during the familiarization period. This subjective feedback was essential in assessing their comfort. Additionally, one of the authors, with >20 years of experience in running research and coaching, monitored the participants' running form, focusing on rhythm, stride, and ground contact patterns. However, recognizing that these are primarily technical measures, the subjective assessments from the participants were crucial in providing a comprehensive evaluation of comfort. This combined approach ensured that any discomfort was identified and addressed before proceeding with the second laboratory session. The participants were equipped with neutral running shoes (Esperanzer, Mizuno Corporation, Japan) to eliminate potential footwear effects on metabolic state (Hanson et al., 2011) and to ensure that neither foot strike pattern was promoted (Squadrone et al., 2015). In the second laboratory session, each participant arrived at the laboratory after fasting for at least 3 h and refrained from exercising before data collection. Each participant was allowed to warm up (treadmill running at a self‐selected speed and free stretching) for several minutes, as needed after the EMG electrode was attached. Subsequently, they were instructed to perform two 3‐s isometric maximal voluntary contractions (MVCs) using a dynamometer (Biodex System 4, Biodex, New York, USA). After placing the reflective markers (Nobby Tech, Tokyo, Japan) on the right lower limb with a shoe, the participant began data collection by standing quietly on the treadmill for 10 min to record baseline VO_2_. Subsequently, the participants completed a warm‐up running on a treadmill at 15 and 19 km/h until they felt they had enough. The resulting total time for warm‐up running was 6:04 ± 5:26 mm:ss. The participants ran on the treadmill at 15 and 19 km/h for 10 s to determine the preferred foot strike pattern. Subsequently, the participants ran using each foot strike pattern for 5 min at 15 and 19 km/h with a 5‐min rest interval. The order of the foot strike patterns was randomized, whereas the running speeds were fixed in a sequential order of 15 km/h, followed by 19 km/h. Each participant rested until the minute VO_2_ returned to within 0.02 L/min of the baseline value (Gruber et al., 2013). Respiratory gas, EMG, and motion capture data were recorded throughout the tests (Figure 1). The room temperature set by the air conditioner was similar for all the participants.

### 2.3 Respiratory gas

Respiratory gas exchange data were continuously measured on a breath‐by‐breath basis using a gas analyzer (AE‐310S, Minato Medical Science, Osaka, Japan). Almost all previous studies that found a difference in running economy between foot strike patterns have calculated average VO_2_ value from the last ≥2 min of data (Gruber et al., 2013; Melcher et al., 2017; Moore et al., 2019; Ogueta‐Alday et al., 2014). In contrast, many studies that detected no difference have calculated these values from the last 1 min of data (Ardigo’ et al., 1995; Di Michele & Merni, 2014; Perl et al., 2012; Stearne et al., 2016). Therefore, the average VO_2_ values were calculated from the last 2 min of VO_2_ data acquired during 5 min of running under each condition using the included software. Subsequently, each average VO_2_ value was divided by the participant's body weight and converted into a mass‐specific value. Energy expenditure (EE) was calculated from VO_2_ and VCO_2_ using Elwyn's energy calculation formula (Bursztein et al., 1989). One participant was unable to complete the 5‐min run at 19 km/h; thus, the participant's data were excluded from subsequent analyzes.

### 2.4 Motion capture

Marker positions were tracked at 100 Hz using a motion capture system (Raptor‐12, Motion Analysis, CA, USA). Reflective markers were attached to the head of the second and fifth metatarsals, calcaneus, lateral malleolus, lateral femoral condyle, and greater trochanter of each participant's right lower limb. The marker data were smoothed by applying a bidirectional second‐order low‐pass Butterworth filter. The cutoff frequency was determined for each marker by residual analysis (Winter, 2009). The residual analysis was performed from 0 to 8 Hz in 0.5‐Hz steps. The plane comprising the anterior–posterior and up‐down directions of the treadmill was defined as the sagittal plane. The knee and ankle joint angles on the sagittal plane were calculated from marker coordinate data of the lateral malleolus, lateral femoral condyle, and greater trochanter and those of the head of the second metatarsal, calcaneus, lateral malleolus, and lateral femoral condyle, respectively. The marker coordinates data of the calcaneus and head of the fifth metatarsals among up‐down directions were used to judge whether the right foot was grounded. In this study, the phase when both the markers of the calcaneus and head of the fifth metatarsal are higher than corresponding thresholds was defined as the swing phase, and another phase was defined as the stance phase. Each threshold was calculated by adding three standard deviations to the mean value of each marker height at standing before running in 0.25 s. The preferred foot strike pattern at each speed was determined by using motion capture. In five cycles from the time when the running speed reached the target speed, it was calculated which part (that is, the calcaneus [rearfoot] or head of the fifth metatarsal [forefoot]) performed the initial contact. The higher ratio of foot strike pattern was defined as the preferred foot strike pattern at each speed. The 10‐cycle joint angle data from 3 min after the start of the run were analyzed. The duration of the stance and swing phases, that of the running cycle, and knee and ankle joint angles at initial contact were averaged for 10 cycles. Each foot strike pattern for 10 cycles was analyzed, and the achievement rate of the specified foot strike pattern was calculated.

### 2.5 Electromyographic activity

Surface EMG activities of the TA, MG, LG, and Sol muscles of the right lower limb were measured using single differential Trigno standard EMG sensors (Trigno; Delsys Inc., Boston, MA, USA; fixed 1‐cm interelectrode distance). The sensors were placed on the skin over the belly of the TA, MG, LG, and Sol muscles after carefully cleaning the skin with alcohol according to the guidelines of surface EMG recordings (Freriks et al., 1999). The EMG signals were bandpass filtered at 20–450 Hz, amplified (×909) using a wireless EMG system (Trigno Wireless EMG System, Delsys Inc., Boston, MA, USA), and stored at 1 kHz using a 16‐bit analog‐to‐digital converter (PowerLab 16/35, ADInstruments, Sydney, Australia). During the MVC trial, the participant lay prone on a seat reclined horizontally. The right ankle was positioned at 0° (neutral), and the right foot was tightly fixed to the plate of a dynamometer (Biodex System 4, Biodex Medical Systems, Shirley, NY, USA). The dynamometer's rotational axis was aligned with the anatomical axis of ankle dorsiflexion and plantar flexion. The root mean square (RMS) for 0.25 s of each muscle during MVC was defined as the MVC value of each muscle. The 10‐cycle EMG data from 3 min after the start of the run were analyzed. The 10‐cycle average of RMSs of each muscle during each of the stance and swing phases was calculated and expressed as % of the MVC value.

### 2.6 Synchronization

For each running trial, the examiner visually observed the speed display of the treadmill and manually operated an in‐house LabVIEW (National Instruments, Austin, TX, USA) algorithm when the set speed was reached. The program outputs transistor‐transistor logic pulses to the analog input of PowerLab and the motion capture system using a DAQ device (NI USB‐6008, National Instruments, Austin, TX, USA), and the time synchronization is based on the timing of the rising edge of the pulse. The VO_2_ data were also output to PowerLab from the analog output of the gas analyzer. Based on the output data, the range for calculating the average VO_2_ values in the included software of the gas analyzer was adjusted to be strictly within the range of the last 2 min of each condition. In one condition (forefoot strike at 19 km/h) for one participant, a problem occurred when the cable disconnected from the equipment in the last 15 s of the trial; therefore, 15 s was not included in their analysis. The resulting calculation time range for the average VO_2_ value was 1:56 ± 0:02 mm:ss.

### 2.7 Statistical analyses

A two‐way analysis of variance with repeated measures (two foot strike patterns × two running speeds) was performed to test for differences in the durations of the stance and swing phases and running cycles, knee and ankle joint angles at initial contact, RMS of TA, MG, LG, and Sol muscles, and VO_2_ using statistical software (SPSS Statistics 21, IBM Japan, Tokyo, Japan). When the data of a participant were not completed for a given index (e.g., TA RMS), the data of the participant were excluded from the statistical analysis of the index. The level of statistical significance for all comparisons was set at *p* < 0.05.

## 3 RESULTS

**FIGURE 2:**
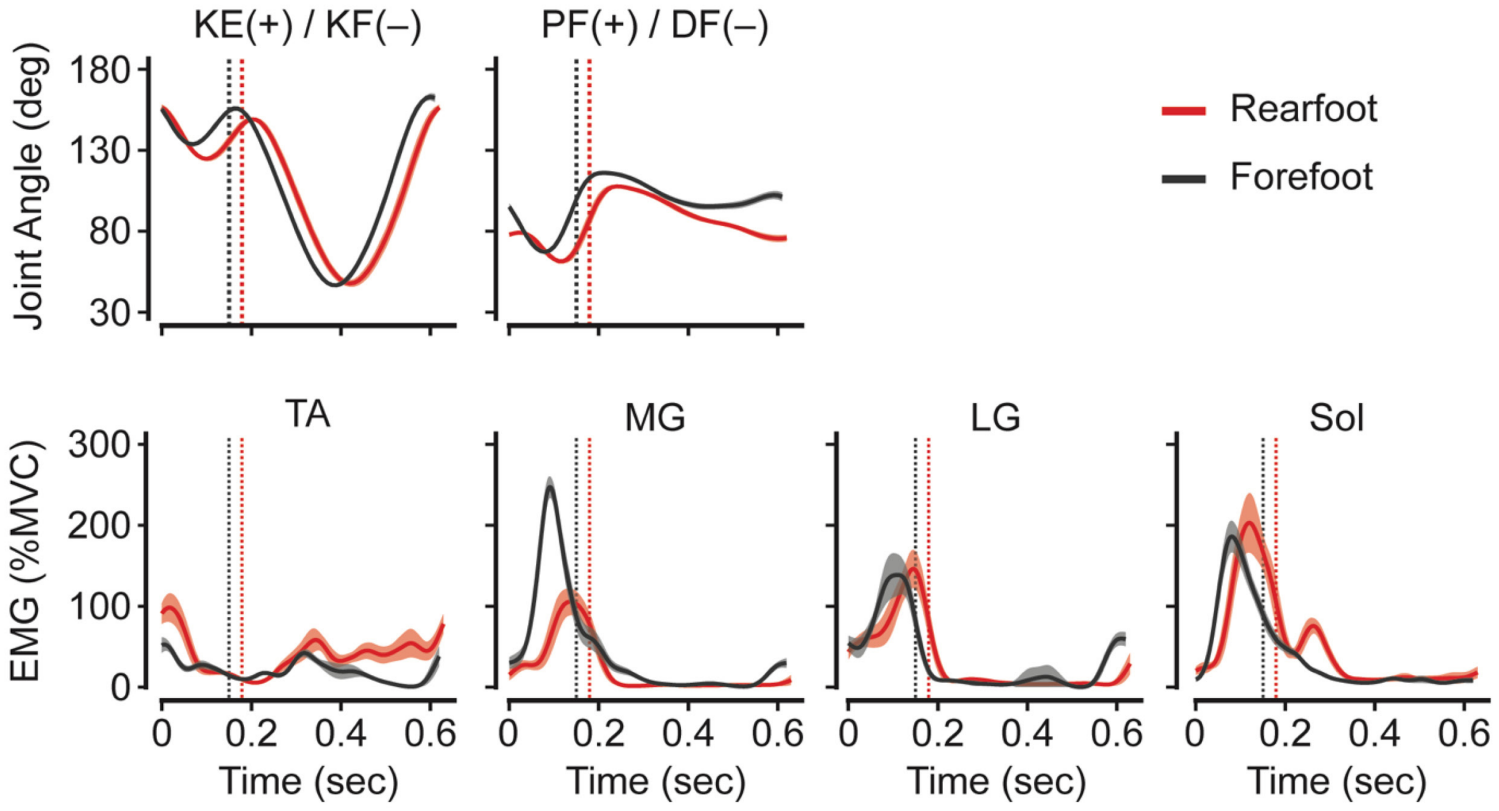
A typical example of joint angles and amplitudes of electromyographic (EMG) activities during running at 19 km/h. Black and red lines with dark gray and pink shades represent ensemble averages with standard deviations for forefoot and rearfoot strike data, respectively. A dotted line represents an averaged transition point from the stance to the swing phase. DF, dorsiflexion; KE, knee extension; KF, knee flexion; LG, lateral gastrocnemius; MG, medial gastrocnemius; MVC, maximal voluntary contraction; PF, plantar flexion; Sol, soleus; TA, tibialis anterior.

A typical example of the knee and ankle joint angles and EMG activities at 19 km/h is shown in Figure 2. The knee joint appeared to be more flexed at initial contact with a forefoot strike than with a rearfoot strike. The TA and MG activities appeared to be lower and higher, respectively, during the stance phase with a forefoot strike than with a rearfoot strike. These observations were confirmed in the subsequent analysis.

**FIGURE 3:**
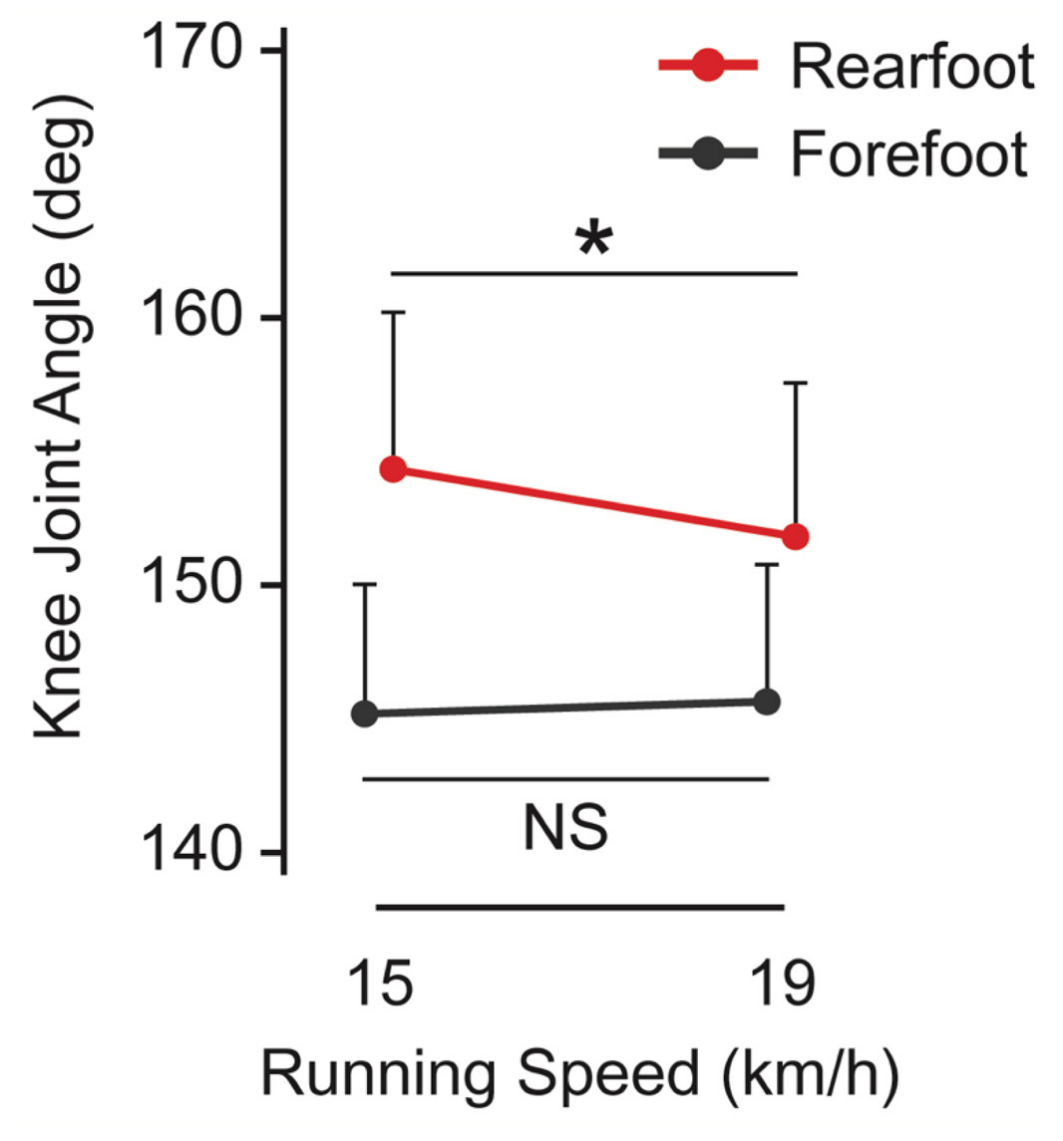
Knee joint angle at foot contact during running. Participants ran at 15 and 19 km/h with the forefoot (black) and rearfoot (red) strikes. An error bar represents a standard deviation. *p < 0.05, indicating a significant simple effect of running speed with a rearfoot strike. NS, indicating no significant simple effect of running speed with a forefoot strike.

The proportion of forefoot strikers at 15 and 19 km/h among all participants was 86.7% and 93.3%, respectively. The achievement rate of the specified foot strike pattern among all conditions was 92.1%. All results of the main effect and interaction on each kinematic parameter are shown in Table 1. The forefoot striking was accompanied by a shorter stance time (*p* = 0.019) and longer swing time (*p* = 0.0427). The higher‐speed running was accompanied by a shorter stance time (*p* = 0.010) and a swing time (*p* = 0.013). There was no significant interaction in the duration of phases and cycles. There was a significant interaction on the knee joint angle (*p* = 0.003). The knee joint angle with a rearfoot strike at 19 km/h was more flexed than that at 15 km/h (*p* = 0.001), although there was no significant difference in the knee joint angle with a forefoot strike between 15 and 19 km/h (Figure 3).

**TABLE 1:** Kinematic parameter results.

Measurements	Foot strike	Speed (km/h)	Mean	±	SD	df	Main effect				Interaction	
							Foot strike		Speed			
							*F*	*p* value	*F*	*p* value	*F*	*p* value
Duration (s)												
Stance	Forefoot	15	0.15	±	0.01	14	7.0	0.019	8.8	0.010	2.8	0.119
		19	0.13	±	0.01							
	Rearfoot	15	0.16	±	0.01							
		19	0.14	±	0.01							
Swing	Forefoot	15	0.52	±	0.02	14	5.0	0.043	8.1	0.013	2.3	0.154
		19	0.46	±	0.02							
	Rearfoot	15	0.51	±	0.03							
		19	0.48	±	0.02							
Joint angle (°)												
Knee	Forefoot	15	151.0	±	4.1	14	7.4	0.016	6.7	0.021	12.5	0.003
		19	150.8	±	4.6							
	Rearfoot	15	154.9	±	5.4							
		19	152.1	±	4.9							
Ankle	Forefoot	15	88.0	±	4.1	14	117.0	0.000	13.2	0.003	1.0	0.327
		19	89.1	±	5.5							
	Rearfoot	15	78.0	±	2.6							
		19	80.2	±	3.9							

Abbreviations: df, degree of freedom; SD, standard deviation.

**FIGURE 4:**
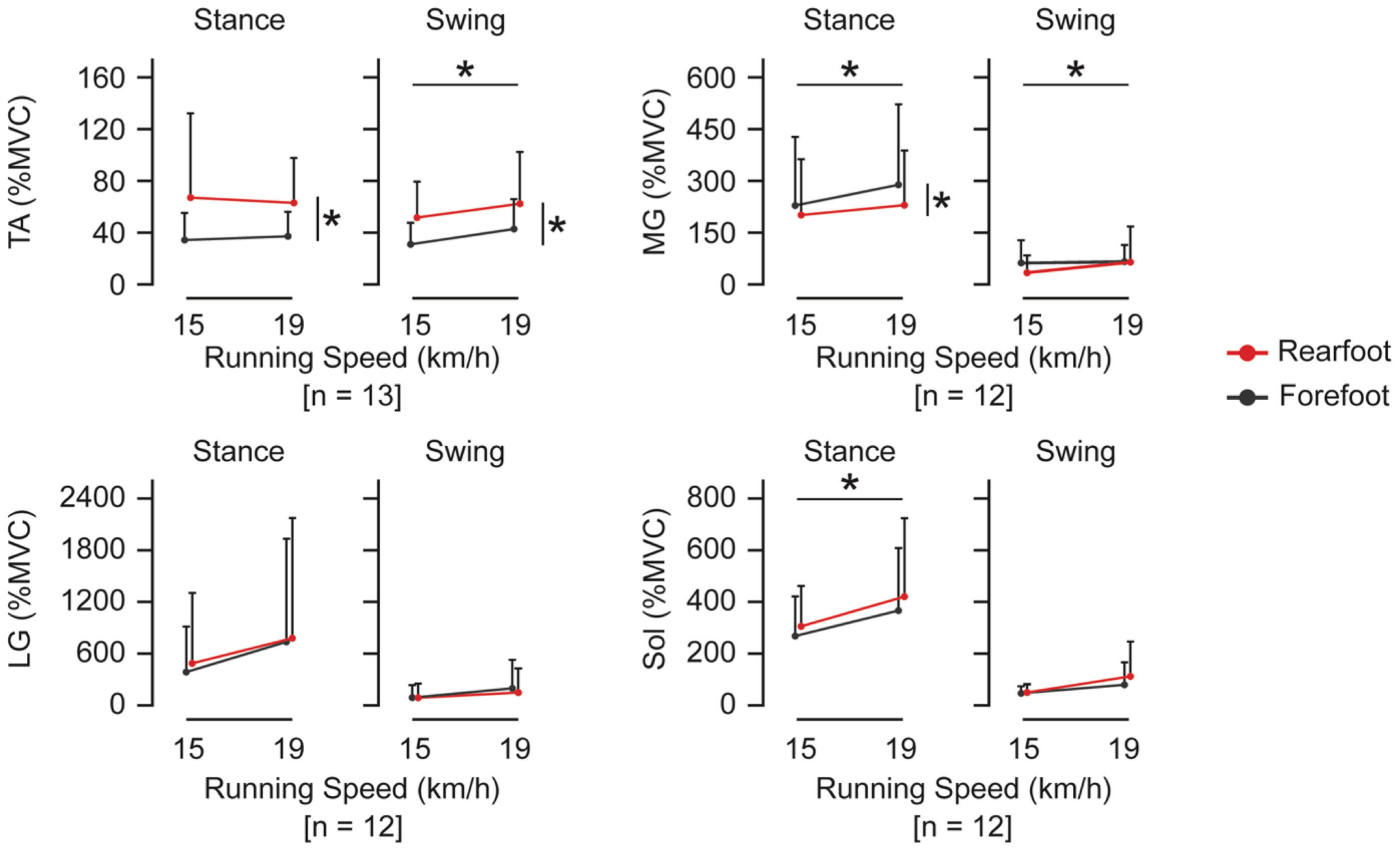
Electromyographic (EMG) activities during the stance and swing phases during running. Participants ran at 15 and 19 km/h with the forefoot (black) and rearfoot (red) strikes. An error bar represents a standard deviation. LG, lateral gastrocnemius; MG, medial gastrocnemius, MVC, maximal voluntary contraction; *n*, the number of analyzed participants; Sol, soleus; TA, tibialis anterior. *p < 0.05, indicating a significant main effect of foot strike pattern or running speed.

All results of the main effect and interaction on each EMG RMS are shown in Table 2. The forefoot striking was accompanied by a lower TA RMS (*p* = 0.029) and MG RMS (*p* = 0.035) during the stance phase and a TA RMS (*p* < 0.001) during the swing phase (Figure 4). The higher speed running was accompanied by a higher MG RMS (*p* < 0.001) and Sol RMS (*p* = 0.031) during the stance phase and a higher TA RMS (*p* = 0.006) and MG RMS (*p* = 0.027) during the swing phase (Figure 4). There was no significant interaction on the RMSs of each muscle during each phase.

**TABLE 2:** All electromyographic (EMG) activity, VO_2_, and energy expenditure (EE) results.

Measurements	Phase	df	Main effect				Interaction	
			Foot strike		Running speed			
			*F*	*p* value	*F*	*p* value	*F*	*p* value
EMG								
TA	Stance	12	6.2	0.029	0.0	0.939	0.3	0.596
MG		11	5.8	0.045	27.0	0.000	3.5	0.090
LG		11	1.2	0.301	1.7	0.217	0.6	0.464
Sol		11	0.8	0.396	6.1	0.031	0.1	0.827
TA	Swing	12	20.1	0.001	11.0	0.006	0.1	0.811
MG		11	1.9	0.194	6.5	0.027	1.5	0.246
LG		11	0.5	0.486	2.6	0.139	0.9	0.371
Sol		11	1.0	0.331	3.7	0.079	0.6	0.468
VO_2_		14	0.1	0.797	556.4	0.000	2.4	0.143
EE		14	0.0	0.978	505.2	0.000	0.7	0.423

Abbreviations: df, degree of freedom; EE, energy expenditure; LG, lateral gastrocnemius; MG, medial gastrocnemius; Sol, soleus; TA, tibialis anterior.

**FIGURE 5:**
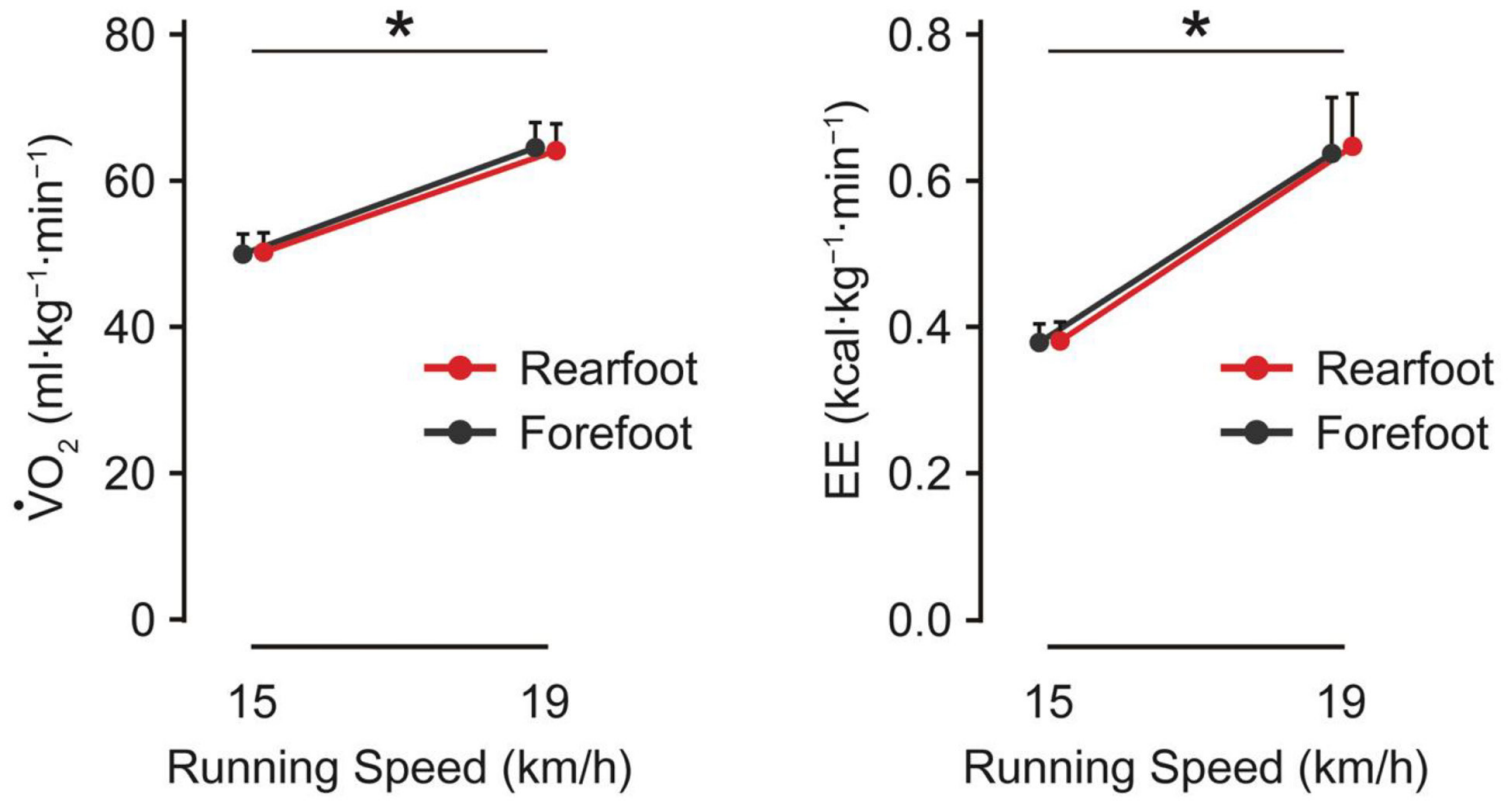
Group average data (*n* = 15) of VO_2_ and energy expenditure (EE). Participants ran at 15 and 19 km/h with the forefoot (black) and rearfoot (red) strikes. An error bar represents a standard deviation. *p < 0.05, indicating a significant main effect of running speed.

The higher running speed was accompanied by a higher VO_2_ and EE (*p* < 0.001). There was no significant interaction on the VO_2_ and EE (Figure 5 and Table 2).

## 4 DISCUSSION

The main findings of this study were that during the stance phase of running at 15 and 19 km/h, the activations of the TA and MG muscles were lower and higher, respectively, with a forefoot strike than with a rearfoot strike. The foot strike pattern did not influence the oxygen uptake.

The results of activations of the TA and MG muscles in this study were consistent with those of many previous studies at speeds of ≤16 km/h. In particular, some EMG studies have indicated that a forefoot strike leads to plantar flexion dominance during a co‐contraction around the ankle (Landreneau et al., 2014; Valencia et al., 2022), as shown in this study (Figure 3). The results of this study corroborate the relationship between the foot strike pattern and TA and MG muscles at speeds ranging up to 19 km/h. Softened co‐contraction could improve the running economy. Although a previous study reported that more economical runners were associated with a longer duration of coactivation of the rectus femoris and LG muscles (Heise et al., 2008), another study reported that a longer coactivation duration of this pair and other pairs of knee extensor and flexor muscles led to worse oxygen consumption (Moore et al., 2014) at <13 km/h. Therefore, softened coactivation of the MG and TA muscles with a forefoot strike could be expected to contribute to efficient running in this study.

However, the oxygen uptake and EE were not influenced by the foot strike pattern at 15 and 19 km/h. Several previous studies have reported the superiority of running with a rearfoot strike in running economy at <15 km/h (Gruber et al., 2013; Melcher et al., 2017; Moore et al., 2019; Ogueta‐Alday et al., 2014), whereas some studies have found no difference in running economy between foot strike patterns even at <15 km/h (Ardigo’ et al., 1995; Di Michele & Merni, 2014; Perl et al., 2012; Roper et al., 2017; Stearne et al.,  2016). Although a previous study reported the superiority of non‐rearfoot strikers in running economy at 13.5 km/h, the age and 10‐km best record of rearfoot strikers were, on average, >12 years and >2 min slower, respectively, than those of non‐rearfoot strikers (Santos‐Concejero et al., 2014). Such a between‐subject comparison includes factors other than the foot strike pattern for the running economy. These studies suggest that a rearfoot strike would be equal to or rather better for the running economy than a non‐rearfoot strike at <15 km/h. However, the effect of the foot strike pattern on running economy would depend on running speed. For example, a previous study reported that the running economy of non‐rearfoot strikers was similar to that of rearfoot strikers at 15 km/h. However, the latter was better than the former at <15 km/h (Ogueta‐Alday et al., 2014). The results of this study at ≥15 km/h were seen as an extension of the relationship between foot strike pattern and running economy at ≤15 km/h.

The mechanical constraints with a forefoot strike could be preferable to those with a rearfoot strike at elite marathoners' speeds. The knee joint angle at an initial rearfoot strike was more flexed with increasing running speed from 15 to 19 km/h, while that at a forefoot strike did not change; this result has not been observed at ≤18 km/h (Ahn et al., 2014; Suzuki et al., 2019). Furthermore, the force demand for the triceps surae muscles can change, particularly from approximately 21 km/h. For example, the peak force generation of the Achilles tendon during running decreases as the running speed increases from approximately 21 km/h (Komi, 1990), partly because the contact time decreases (Hayes & Caplan, 2012). Physiologically, a forefoot strike induces a longer Achilles tendon, which indicates larger force generation at initial contact, at least at 18 km/h (Suzuki et al., 2019). In addition, the force generation of the Achilles tendon is more rapid with a forefoot strike than with a rearfoot strike (Komi, 1990). Therefore, a forefoot strike would be adequate for force generation within a short contact time, and a forefoot strike might lead to a better running economy than a rearfoot strike at such speeds. Consequently, it could be assumed that the superiority of a rearfoot strike would decrease with increasing running speed, and a forefoot strike would reverse a position at a certain speed. If the results of this study are interpreted in this context, speeds from 15 to 19 km/h would enter a turning zone in the running economy. However, because the influence of the foot strike pattern on running economy at >19 km/h has not been examined, future studies are warranted to test the assumption of this influence on overall practical speeds.

A limitation of this study is that most of the participants were forefoot strikers who were relatively unaccustomed to a rearfoot strike. An unaccustomed foot strike without long‐term training can lead to a negative bias regarding the running economy. For example, running with a non‐rearfoot strike at <11 km/h is inefficient in oxygen consumption for rearfoot strikers after two 30‐min training sessions for accommodation, whereas this inefficiency decreases to an insignificant level after a 14‐week intervention (Ekizos et al., 2018). The rearfoot strike had a bias disadvantage regarding the running economy because there were few rearfoot strikers in the present study. For a fair comparison, future studies are encouraged to recruit the same number of forefoot and rearfoot strikers.

In conclusion, the foot strike pattern has no clear effect on the oxygen uptake when running at 15 and 19 km/h. However, the forefoot strike leads to plantar flexion dominance during the coactivation of the MG and TA muscles, which are agonist and antagonist muscles for plantar flexion, respectively, during the stance phase.

## PERSPECTIVES AND SIGNIFICANCE

Previous studies reported the superiority of a rearfoot strike at <15 km/h (Gruber et al., 2013; Melcher et al., 2017; Moore et al., 2019; Ogueta‐Alday et al., 2014), and the results of the study suggest that the forefoot strike has no advantage in running economy over the rearfoot strike at ≤19 km/h. The speed of 19 km/h is higher than the average speed of the top 50% of athletes at the men's 2017 World Championship marathon (Hanley et al., 2019). Although some coaches assume the inferiority of the rearfoot strike for a long‐distance performance (Abran et al., 2022), the forefoot strike should not be recommended, at least for recreational marathoners, from the perspective of performance.

## AUTHOR CONTRIBUTIONS

T.S. and R.K. conceived and designed research; S.K., K.Y., and R.K. performed experiments; S.K. and K.Y. analyzed data; S.K., K.Y., T.S., E.D., Y. K., and R.K. interpreted results of experiments; S.K. prepared figures; S.K., K.Y., T.S and R.K. drafted the manuscript; S.K., K.Y., T.S., E.D., Y.K., and R.K. edited and revised manuscript; S.K., K.Y., T.S., E.D., Y.K., and R.K. approved the final version of manuscript.

## FUNDING INFORMATION

This work was supported by a Research Collaboration Grant from Kanagawa University (to R.K.) and JSPS KAKENHI Grant Number JP22K17703 (to T.S.).

## CONFLICT OF INTEREST STATEMENT

The authors have declared that no competing interests exist.

## ETHICS STATEMENT

This study was approved by the Human Research Ethics Committee at Kanagawa University (2019‐10‐6).

## CONSENT

Each participant provided written informed consent prior to participation.

## Data Availability

The data that support the findings of this study are available from the corresponding author upon reasonable request.
